# Individual Patterns and Temporal Trajectories of Changes in Fear and Pain during Exposure In Vivo: A Multiple Single-Case Experimental Design in Patients with Chronic Pain

**DOI:** 10.3390/jcm11051360

**Published:** 2022-03-01

**Authors:** Jente Bontinck, Marlies den Hollander, Amanda L. Kaas, Jeroen R. De Jong, Inge Timmers

**Affiliations:** 1 Department of Rehabilitation Sciences and Physiotherapy, Ghent University, 9000 Ghent, Belgium; jente.bontinck@ugent.be; 2 Pain in Motion International Research Group, Department of Physiotherapy, Human Physiology and Anatomy, Faculty of Physical Education & Physiotherapy, Vrije Universiteit Brussel, 1090 Brussels, Belgium; 3 Department of Rehabilitation Medicine, Maastricht University, 6211 LK Maastricht, The Netherlands; m.hollander@adelante-zorggroep.nl (M.d.H.); jeroen.dejong@integrin.nl (J.R.D.J.); 4 Adelante Centre of Expertise in Rehabilitation and Audiology, 6430 AB Hoensbroek, The Netherlands; 5 Department of Cognitive Neuroscience, Maastricht University, 6229 EV Maastricht, The Netherlands; a.kaas@maastrichtuniversity.nl; 6 Intergrin Academy for Specialized Healthcare, 6167 AC Geleen, The Netherlands

**Keywords:** chronic pain, exposure in vivo, pain-related fear, rehabilitation, chronic low back pain, complex regional pain syndrome

## Abstract

Exposure in vivo (EXP) is an effective treatment to reduce pain-related fear and disability in chronic pain populations. Yet, it remains unclear how reductions in fear and pain relate to each other. This single-case experimental design study attempted to identify patterns in the individual responses to EXP and to unravel temporal trajectories of fear and pain. Daily diaries were completed before, during and after EXP. Multilevel modelling analyses were performed to evaluate the overall effect. Temporal effects were scrutinized by individual regression analyses and determination of the time to reach a minimal clinically important difference. Furthermore, individual graphs were visually inspected for potential patterns. Twenty patients with chronic low back pain and complex regional pain syndrome type I were included. On a group level, both fear and pain were reduced following EXP. Individually, fear was significantly reduced in 65% of the patients, while pain in only 20%. A decrease in fear was seen mostly in the first weeks, while pain levels reduced later or remained unchanged. Daily measurements provided rich data on temporal trajectories of reductions in fear and pain. Overall, reductions in fear preceded pain relief and seemed to be essential to achieve pain reductions.

## 1. Introduction

Chronic pain is characterized by a complex interaction between physical and psychosocial factors, and therefore remains a therapeutic challenge [1]. Psychosocial factors have been recognised as important contributors to the development and maintenance of chronic pain and related disability. A major role is played by pain-related fear [2]. After an acute injury, it is a beneficial protection mechanism to fear and avoid activities that are associated with pain and potential further damage. Yet, this behaviour becomes maladaptive when it persists into the chronic stage [3]. Avoidance of daily activities results in functional deterioration, contributes to more pain and increases the fear of (re)injury [4]. This vicious circle is described as the fear-avoidance model [2].

Pain-related fear has been considered an important contributing factor in chronic musculoskeletal pain populations [2,5,6]. For instance, in patients with chronic low back pain (cLBP), fear contributes more to disability than pain intensity [7] and is associated with poorer recovery [8,9]. Furthermore, the recovery of patients with complex regional pain syndrome type I (CRPS-I) is adversely influenced by pain-related fear [10,11]. In fact, both populations showcase high similarities in fear and its association with disability [12].

Consequently, it is important to address pain-related fear in the rehabilitation of patients with chronic pain. Exposure in vivo (EXP) is a cognitive-behavioural treatment that stimulates patients to perform threatening movements and activities, in order to modify their expectations about movement and injury, and to reduce their avoidance behaviour [13]. Combining exposure with pain education teaches patients that their disability is self-manageable and their pain is not necessarily a reliable indicator of injury [14]. EXP has been demonstrated to be effective in patients with cLBP [15,16,17], CRPS [18,19], and other pain types [20,21]. Although EXP decreases fear and disability in most patients, approximately 40–60% does not respond with clinically relevant changes in pain experiences [17,22]. Importantly, this treatment does not explicitly target pain levels. The primary intention is to lower pain-related fear and consequently disability, as they show a strong association unaffected by pain intensity [4]. Conversely, fear does mediate the associations between pain and disability [9]. Fear influences the report of pain [23], but is also identified as a consequence rather than a precipitating factor of pain [24]. These findings suggest an unique but also complicated relationship between fear and pain. It has been anecdotally reported that during EXP fear is reduced first and then followed by reductions in pain, but this has not been formally investigated. It also remains unclear whether reductions in fear are a prerequisite for pain relief. 

Therefore, the primary aim of this study was to examine the temporal relationship between changes in pain-related fear and pain intensity during EXP in patients with chronic pain. Daily diaries were used to scrutinise the chronology of treatment effects and to identify individual patterns. These insights could help clinicians to improve patient-tailored treatment approaches. Our hypothesis was that decreases in fear during EXP would precede pain relief.

## 2. Materials and Methods

### 2.1. Study Design

A sequential single-case experimental design (SCED) was used in this study, in which several outcomes per participant were repeatedly assessed throughout different phases [25]. Participants completed daily repeated measures during a baseline period (phase A), an intervention phase (phase B), and an immediate post-intervention period (phase C). In addition to the diaries, online questionnaires were completed at baseline and post-treatment.

### 2.2. Procedures

This SCED study is part of a larger study investigating effects of EXP on chronic pain, “BrainEXPain”. BrainEXPain was approved by the Medical Ethical Committee of Maastricht University Hospital/Maastricht University (MUMC+/UM). The protocol is registered at ClinicalTrials.gov [NCT02347579]. Previous papers of the “BrainEXPain” project focused on fMRI [22,26], in relation to pain-related questionnaires. The results of the diaries have not been described yet.

**Figure 1:**
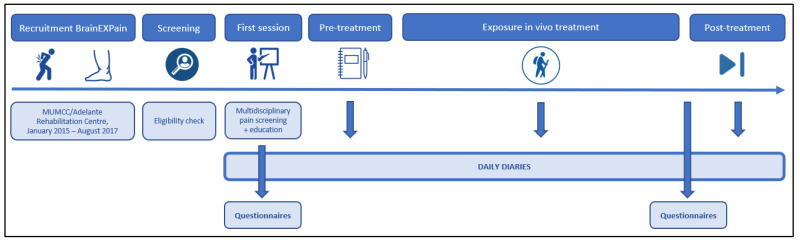
Timeline study procedure.

Patients were recruited via the department of Rehabilitation Medicine at MUMC+/Adelante Rehabilitation Centre, between January 2015 and August 2017. They were referred by a physiatrist to a multidisciplinary screening procedure and were requested to fill out an online screening questionnaire (Qualtrics, Provo, UT, USA). Eligible patients, for whom pain-related fear was suspected to be a major contributing factor, were briefed on the study procedure, daily diaries, and treatment approach, and were asked to sign the informed consent. If both the multidisciplinary team and the patient gave a green light, information about EXP and an introduction to pain education was provided. Prior to the first study visit in which an MRI scan was performed, patients were requested to fill out online questionnaires and to start with filling out daily diaries (Qualtrics, Provo, UT, USA) for five consecutive days or more, which resulted in a pseudo-randomized baseline period. Three days prior to the first treatment session, patients were requested to start completing daily diaries again and to continue throughout the whole treatment period. Daily diaries were consistently sent out at 9:00 am. The online questionnaires were repeated post-treatment and the patients were asked to continue filling out the diaries for another two weeks (Figure 1).

### 2.3. Participants

The study included patients with cLBP as well as patients with CRPS, between 18 and 65 years old. To be included, patients had to experience non-specific LBP for at least 6 months or to be diagnosed with complex regional pain syndrome type-I (CRPS-I) based on the Budapest criteria [27]. Patients were referred for EXP if irrational cognitions and pain-related fear was deemed to be a maintaining factor for pain-related disability, by an experienced rehabilitation team (including a physiatrist, a physical therapist, a psychologist, and an occupational therapist). Exclusion criteria were other diagnoses that could explain the symptoms, pregnancy, and serious psychopathology diagnosed with the Symptom Checklist (SCL-90) [28].

### 2.4. Exposure In Vivo Treatment

Exposure in vivo (EXP) is standard care for patients with chronic pain and elevated pain-related fear at MUMC+/Adelante. This treatment exposes patients to feared movements and activities, in order to challenge and adjust their exaggerated expectations related to harm and (re-)injury. EXP aims to modify fear-avoidance beliefs by increasing knowledge about pain, encouraging to perform threatening activities, and challenging patient’s expectations about consequences of movement. A detailed description of the EXP-protocol can be found in Vlaeyen et al. [29]. First, individual threatening activities were identified by using the photograph series of daily activities (PHODA) [30]. Treatment started subsequently with pain education, in which was explained that pain is not an indicator of harm and may persist in a vicious fear-avoidance circle. In the EXP sessions, threatening activities selected from the patient’s completed PHODA were performed to challenge their expectancies and were encouraged to repeatedly perform relevant threatening activities until they no longer intended to avoid them. EXP typically consist of 16 sessions, but the number could be adapted based on clinicians’ and patient’s decision.

### 2.5. Outcomes

#### 2.5.1. Daily Measures

Daily levels of pain intensity and self-reported fear of three personally relevant daily-life movements/activities were assessed using electronic diaries. These individually tailored activities were selected based on a ranking of movements/activities (PHODA) by the participant as being threatening and personally relevant. Participants received a daily reminder to complete a brief questionnaire. Participants were requested to complete the daily assessments from baseline until two weeks after the end of treatment. Due to variations in scheduling, the baseline period differed for each participant and hence was pseudo-randomized. All items were rated on a visual analogue scale (VAS) ranging from 0 to 100. The daily questions are described in Table 1. Diaries have been shown to be sensitive to capture the effect of exposure in vivo treatment [11,18,31].

#### 2.5.2. Non-Daily Questionnaires

Online questionnaires were filled out at baseline and post-treatment. The following standardised questionnaires were included: Pain Disability Index [32], photograph series of daily activities (PHODA) for low back [33], upper [34], or lower extremities [35], Tampa Scale of Kinesiophobia (TSK) [36], Pain Catastrophizing Scale (PCS) [37], Hospital Anxiety and Depression Scale (HADS) [38], Pain Vigilance and Awareness Questionnaire (PVAQ), Short-Form McGill Pain Questionnaire (SFMPQ) [39], and the Resilience Scale (RS) [40]. Participants were also asked to score their pain on a standard 11-point numeric rating scale from 0 (“no pain”) to 10 (“most pain imaginable”) [41]. An average pain score was calculated; combining the current pain, pain from the night before and the worst, the best, and average pain in the last week.

### 2.6. Statistical Analyses

Initially, descriptive statistics of baseline characteristics, including the average fear and pain levels, were examined. Our statistical analyses of daily diary data were conducted with the MultiSCED app (http://34.251.13.245/MultiSCED/; 1 October 2021). MultiSCED has been developed in R to provide the possibility to investigate intervention effects at the individual level and to combine SCED data across cases through multilevel modelling [42]. By creating a multilevel model, the strong internal validity of monitoring a single case can be extended to estimate overall treatment effects [43]. To evaluate the overall changes in pain-related fear and pain intensity, the pre- and post-treatment daily data were compared on a group level. The significance level was set at α = 0.05, 95% CI. Missing data was handled according to the randomized-marker method, in which all days the diary was not completed were displayed as “NA” (not applicable) [44,45].

Subsequently, individual data were examined to identify individual patterns and to unravel interactions between pain-related fear and pain intensity. Individual regression analyses were performed, comparing baseline (phase A) and post-intervention results (phase C) to examine the effect of EXP per individual.

In order to investigate when treatment effects occur during the intervention:

(1) Different phases were created to be used in a sliding window approach. We started comparing baseline data (phase A) with all intervention and post-intervention data (Phase B + C). Afterwards, we systematically added one week to phase A to obtain a timeline for treatment effects (e.g., Phase A + 1w), until phase B consists of less than five measurements.
(2) The time to reach the minimal clinically important difference (MCID) was scrutinized. To unravel the relevance of EXP to patient care, it is useful to not only focus on statistically significant changes, but also on clinically important differences. Even if the statistical result is not significant, the patient might still feel meaningful pain relief or physical improvement due to treatment. A reduction of 30% in pain intensity on a 11-point numeric rating scale has been considered as a MCID in chronic pain populations [46,47]. Therefore, the 30% cut-off value, starting from the baseline average, for pain intensity and for pain-related fear was calculated for each patient. The moment the patient scored lower than this value for at least three consecutive days, was considered as the point of MCID.
(3) Individual visual graphs and descriptive values were explored to divide the patients into clusters based on their response to EXP. Patterns in temporal changes were analysed within these clusters and compared to the results of the sliding window approach, the time to reach the MCID and the differences between baseline and post-intervention questionnaires.

Pre- and post-intervention questionnaires were analysed using SPSS (version 27). First, normality was checked with the Shapiro-Wilk test. Treatment effects of normally distributed variables were analysed with the paired Student’s *t*-test and the paired Wilcoxon-test was used for non-normally distributed variables.

At last, moderator variables for treatment effectiveness were also exploratively scrutinized, by including them one by one in the pre–post multilevel model (e.g., gender, age, population, and the baseline results of the non-daily questionnaires).

## 3. Results

### 3.1. Participant Characteristics

**Figure 2:**
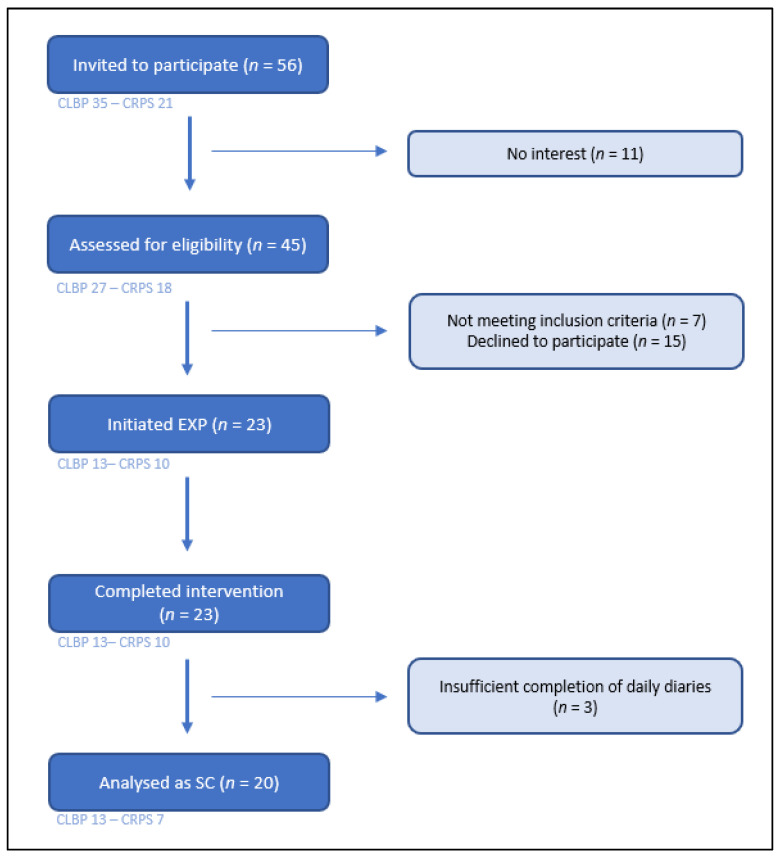
Flow chart of recruitment process. CLBP = chronic low back pain; CRPS = complex regional pain syndrome type I; Exp = exposure in vivo treatment; SC = single case subject.

The recruitment period resulted in 38 included patients in BrainEXPain, of which 23 initiated and completed EXP. Based on the completeness of the daily diaries (minimal duration of treatment of five weeks with at least 40% of the daily diaries completed), 20 patients were analysed as single-cases in this study (Figure 2), including thirteen patients with cLBP and seven with CRPS. The baseline demographic characteristics and most relevant reported outcomes can be found in Table 2. Detailed information on baseline scores for all questionnaires can be accessed in Supplementary materials. 

### 3.2. Treatment Characteristics

Patients initially received two sessions per week, which was reduced to one session per week in the generalisation phase. Total treatment duration ranged from 5 to 14 weeks, with an average of 9 weeks, which was determined by a common decision between the therapist and the patient (Table 3).

### 3.3. Diary Completion

An overview of the diary completion per patient and period can be found in Table 3. Baseline data contained 3 to 12 measures scattered throughout the five months prior to treatment, of which at least the three days right before the start of treatment were acquired. Completion during treatment had an average of 72.5%. Post-intervention data varied between 0 and 19 measures. Unfortunately, eleven patients did not complete the diaries after finishing treatment. When no follow up data was available, the last seven measurements during treatment were utilized for further analyses.

### 3.4. Multi-Level Modelling of Daily Diary Outcomes

Multi-level analyses of pre- and post-intervention diaries were performed to evaluate overall treatment effects (Table S1). Comparison of the daily measurements in phase A versus phase C showed significant improvements in pain related fear (MD = −29.44; SD = 7.30; t = −4.03; *p* ≤ 0.001) and pain intensity (MD = −9.28; SD = 2.61; t = −3.55; *p* = 0.002) (both outcomes were scored on a scale from 0 to 100).

### 3.5. Descriptive and One-Level Analyses of Daily Diary Outcomes

**Figure 3:**
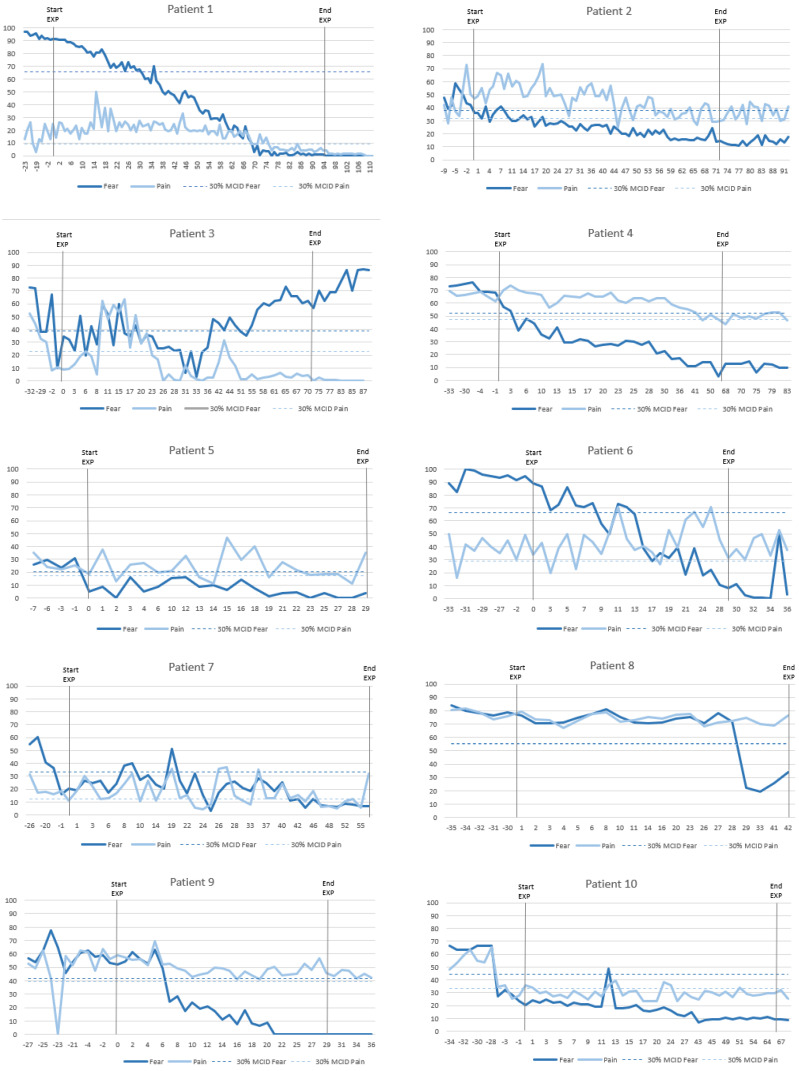

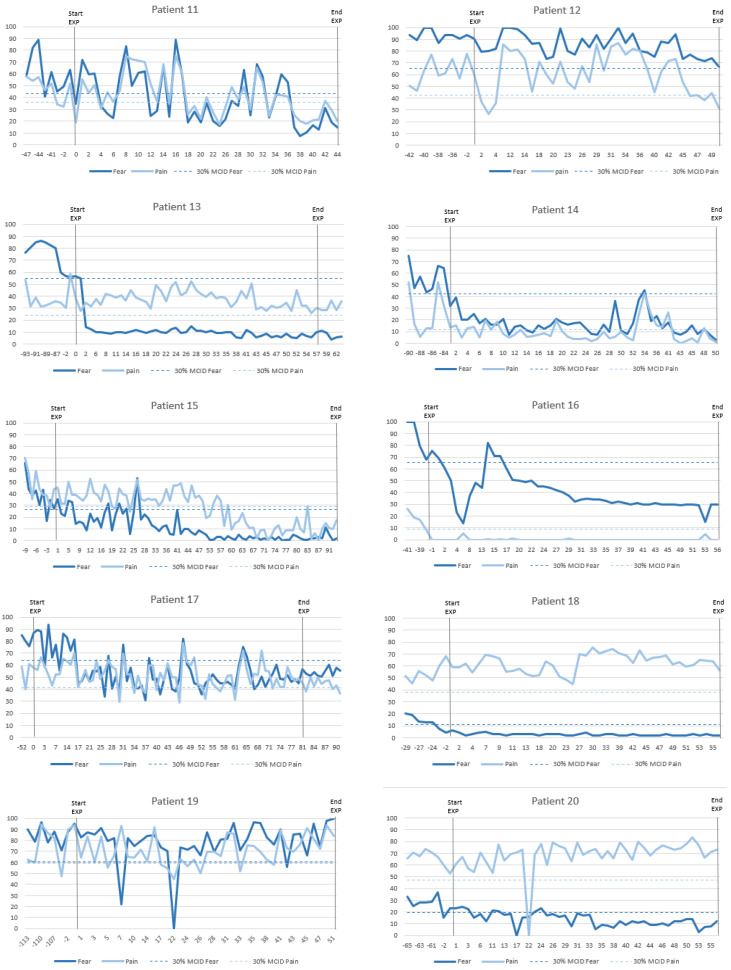
Graphs of daily measurements of pain-related fear and pain intensity.

The evolution of individual daily measurements of pain-related fear and pain intensity across all participants is shown in Figure 3. Visual inspection of these graphs reveals reduction of pain-related fear in almost all patients. Some showed an immediate response to EXP, while others display a delayed reaction. When observing pain intensity, only about half of the patients showed a decrease between baseline and post-intervention. Various patterns can be identified, emphasising that not all patients respond similarly to EXP and strong conclusions based on visual inspection are challenging.

**Figure 4:**
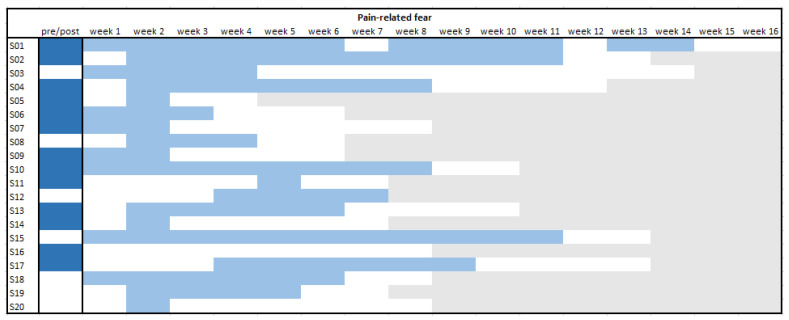
Overview of individual significant reductions per phase for pain-related fear. 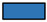 pre–post effect (*p* < 0.05); 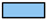 change in trend (*p* < 0.05); 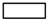 non-significant change; 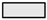 end of EXP. Note. A light blue box means the trend of scores before that specific week was significantly different than the trend of scores after this point. A white box after a light blue box means that that week no big changes occurred anymore.

**Figure 5:**
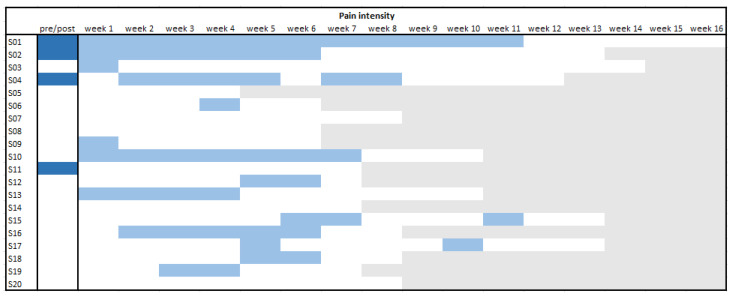
Overview of individual significant reductions per phase for pain intensity. 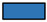 pre-post effect (*p* < 0.05); 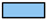 change in trend (*p* < 0.05); 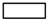 non-significant change; 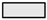 end of EXP. Note. A light blue box means the trend of scores before that specific week was significantly different than the trend of scores after this point. A white box after a light blue box means that that week no big changes occurred anymore.

Therefore, a schematic overview of the one-level analyses is presented in Figure 4 and Figure 5, and detailed statistical information can be found in Tables S2 and S3. First, all daily measurements during baseline phase A were compared with those during the post-intervention phase C. This comparison showcases the effectiveness of EXP for the relevant outcomes. For pain-related fear, the effect of treatment manifests itself in significant reductions in 13 out of 20 patients (65%). By contrast, only four showed a significant reduction for pain between the baseline and post-intervention phase (20%).

Consequently, it was analysed during which week a significant change in trend appeared by comparing all data before the respective week with all further data (i.e., sliding window approach). These results were rather variable, but visual inspection of Figure 4 and Figure 5 shows that patients had earlier and more continuous changes in pain-related fear than in pain intensity. After two weeks, the scores for fear were significantly influenced in 16 of the patients (80%), and for pain, only, in 8 of the patients (40%). Only one patient did not experience a significant change in fear at any point during treatment (even though the overall pre–post comparison did yield a significant difference), while for pain six patients did not show a change in trend at any point.

### 3.6. Time to Reach the Minimal Clinically Important Differences (MCID)

**Figure 6:**
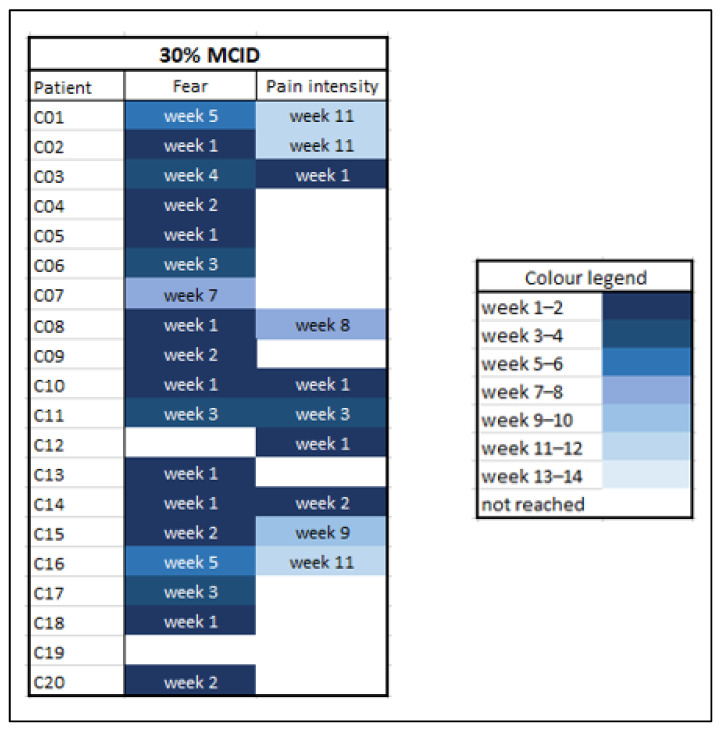
Time to reach the minimal clinically important difference per patient. MCID = minimal clinically important difference, fixed at 30% of the mean baseline score.

An overview of these findings is given in Figure 6. The earlier the clinically meaningful effect occurred, the darker blue the box is coloured. Inspection of this figure quickly shows that the cut-off values for fear were more often and sooner reached than for pain intensity. The fear scores reached the cut-off in all but two patients, while the pain scores reached the MCID in only half of the patients. Of those who reached a MCID for pain, it preceded an MCID in fear in only one patient (10%). The others showed a MCID in fear before or during the same week as an MCID in pain. The range in which the MCID was reached was week 1 to 7 for fear and week 1 to 11 for pain. Additionally, during the first four weeks 15 of the 20 patients reached the MCID for fear, compared to only five for pain.

### 3.7. Visual Inspection of Clusters in Single Cases

**Figure 7:**
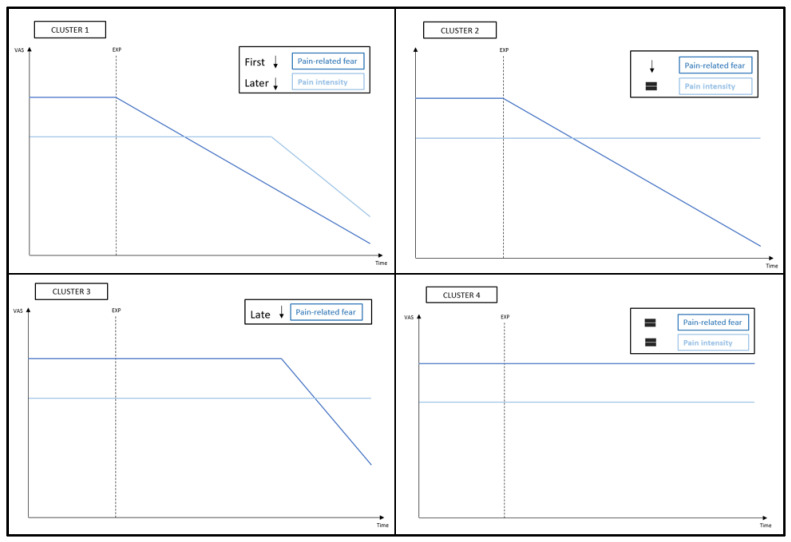
Overview of the clusters.

Closer inspection of the individual graphs in Figure 3 shows that each patient responded differently to EXP. However, different patterns can be recognized and enables us to divide the patients into clusters. Based on the temporal effect on pain-related fear and pain intensity, four clusters could be identified (Figure 7).

Cluster 1: Quick decrease in fear, pain follows

Patients in this cluster showed a decrease in pain-related fear quickly after the start of EXP, while a decrease in pain intensity only occurs later. Seven patients (i.e., 1, 2, 4, 7, 10, 14, and 15) showcase this pattern in greater or lesser extent.

Cluster 2: Decrease in fear, pain unaffected

This pattern is characterized by a decrease in pain-related fear, while the pain remained unchanged. This phenomenon is recognisable in the graphs of five patients (i.e., 5, 6, 9, 13, and 17).

Cluster 3: Late effect in fear

Three patients (i.e., 8, 11, and 16) showed a rather late decrease in pain-related fear, reflecting that they needed multiple treatment sessions and repeated exposure before the effect occurred. While patient 8 has a sudden drop in fear after four weeks, patient 11 had enormous fluctuations until far in treatment, and patient 16 had an initial early drop but relapsed before improvements slowly occurred again. Patient 8 showed no change in pain intensity, while the pain of patient 11 followed the same pattern of the fear and patient 16 described no pain throughout the whole treatment period.

Cluster 4: No clear effect

Based on visual inspection of the graphs, five patients (i.e., 3, 12, 18, 19, and 20) showed little to no change in pain-related fear or pain intensity.

### 3.8. Non-Daily Questionnaires

Before and after treatment the patients filled out online questionnaires. All results can be found in Table S4A,B. All variables were normally distributed. Comparisons of pre- and post-EXP data of all patients showed significant improvements in average pain score (−1.64 ± 2.16; *p* = 0.003), pain-related disability (PDI; −27.4 ± 15.36 SD; *p* < 0.001), pain-related fear (PHODA; −33.33 ± 22.69 SD; *p* < 0.001), fear of movement (TSK; −10.65 ± 7.39 SD; *p* < 0.001), pain catastrophizing (PCS; −12.4 ± 13.08 SD; *p* < 0.001), pain vigilance (PVAQ; −13.75 ± 16.57 SD; *p* = 0.001), pain rating (SFMPQ; −5.4 ± 6.48 SD; *p* = 0.001), and resilience (RS; −7.1 ± 7.45 SD; *p* < 0.001), but not on anxiety and depression (HADS; −2.35 ± 7.17; *p* = 0.16).

Based on the distribution into the four clusters, differences can also be exploratively inspected in questionnaires results. Baseline average pain scores were the highest in cluster 4 compared to the other clusters, while the gain by EXP was the lowest. Baseline PDI scores were higher in cluster 3 and 4, while cluster 2 and 4 showed smaller improvement in disability after treatment. No differences between the clusters were seen in baseline PHODA and RS scores, yet cluster 4 showed again the least improvement in these questionnaires. Other between clusters results were comparable.

### 3.9. Moderating Factors of Treatment Efficacy

In order to evaluate which characteristics influenced the effect of EXP on the outcomes, the baseline scores were included as moderator variables in the pre–post multilevel model (Table S5). Treatment effect on pain-related fear was significantly moderated by gender (*p* = 0.001), showing a lower reduction of fear in women compared to men, but not by age, population, or any of the questionnaire results. None of the baseline characteristics significantly moderated the effect of EXP on pain intensity.

The text continues here. Proofs must be formatted as follows.

## 4. Discussion

The primary aim of this SCED study was to disentangle individual patterns in the temporal effect of EXP on pain-related fear and pain intensity. SCEDs have proven to be valid for demonstrating intervention effectiveness at an individual level and to observe these changes over time [43]. The strong internal validity of monitoring one participant has also been extended by creating a multilevel model, allowing to estimate overall effects across cases. Multilevel analyses revealed that daily fear and pain scores were lower after than before treatment, showing that—overall—EXP had a positive impact on both outcomes. The findings of this study are consistent with previous SCED studies that concluded that EXP had a positive effect on fear and intensity in patients with CRPS-I [18] and cLBP [48]. In addition to the positive group-level effects, individual subject analyses demonstrated that the majority of patients (65%) responded with a reduction in fear, while the effect on pain was more limited (20%). Both the sliding window approach and the time to reach MCID showcased that fear reductions occurred sooner or in absence of pain reductions.

When considering the temporal trajectories by the sliding window approach, it is remarkable that none of the patients showed an improvement in pain without an improvement in fear. This suggests that it is necessary to lower fear to obtain an effect on pain. This fits the idea to predominantly treat fear, as it is more disabling than pain itself [7] and contributes to the maintenance of chronic disability [49]. Disability was reduced by EXP, but it could have been useful to investigate when this effect occurs to understand its relationship with fear and pain. It might be that first new expectations are formed and subsequently fear reduces. Hence, individuals restart to perform formerly threatening activities and eventually their functionality increases. The reduction in pain subsequent to the reduction of fear could be explained by the fact that they share common brain networks [50,51]. Fear of pain involves similar neural circuits as pain perception, including the amygdala, limbic structures, anterior insula, and the adrenomedullary system [52,53,54]. Pain-related fear also recruits distinguishable networks, compared to non-pain related fears [55]. Previous neuroimaging analyses of this project established the involvement of cortico-limbic connectivity in the effect of EXP on pain intensity [22]. In particular, larger decreases in resting-state connectivity between the hippocampus and the posterior medial cortex were associated with larger pain relief and mediated the relationship between catastrophizing and pain. Furthermore, EXP had a positive impact on the medial prefrontal cortex and the right posterior insula, which play a fundamental role in the pain experience [56,57]. The description of pain as an unpleasant sensory and emotional experience [58] and the classification of chronic primary pain [59], highlight the neural and conceptual link between pain and emotion [60]. Pain is inextricably linked with biological, psychological, and socio-cultural factors. Fear, as a strong emotion, can influence pain experiences and reductions in fear can consequentially lead to pain reduction.

Scrutinizing daily assessments showed that each patient responded differently. However, based on the chronology of treatment effects, four patterns could be identified. The pattern of the first cluster—*quick decrease in fear, pain follows*—underscores our hypothesis. The sliding window approach showed that the patients in this cluster benefited already in the first two weeks concerning fear. This pattern is in accordance with the objective of EXP: to target fear and not pain itself. It is also in line with anecdotal reports of clinicians that many patients experience an early eye-opener, after which they manage to “flip the switch”. This group also showed the largest improvement in disability. Their questionnaires results support the daily diaries, which showcases that SCEDs are appropriate to capture individual effects. Concerning the goal of EXP to lower fear, patients in cluster two—*decrease in fear, pain unaffected*—still responded well. Clusters one and two demonstrate an effect in the early phase, what may suggest that pain education and limited exposure sessions already affect fear. Patients in cluster three—*late effect in fear*—needed more EXP sessions before their fear levels distinctly decreased. Remarkable is that this cluster had the highest initial disability and fear. This group may have needed more persuasion before they let go of their avoidance and/or safety behaviours. The difference with cluster one could lay in what they are afraid of. For instance, fear of what can happen during threatening activities could be quickly reduced by EXP, while fear of not being able to handle the pain could be more persistent. However, this remains speculative and would need to be further investigated. Based on the diaries, patients of cluster four—*no clear effect*—did not respond positively. However, questionnaire scores still improved remarkably. Various explanations of why they did not respond as strongly as the others could be considered. First, it cannot be ruled out that their treatment period was too short or delayed treatment effects were not captured within the follow-up period. Delayed effects on pain-related fear have also been seen in youth with chronic pain [61]. Contradictory, short-term EXP might have better results than long-term [17]. Second, while a variety of activities were performed during treatment, only fear of three activities were daily questioned. These may not have been representative enough, given that the complete PHODA showed positive results. Third, it is noticeable that the baseline average pain was higher, while they did not score higher on fear. It may be that patients with higher initial pain benefited less from EXP than patients with higher fear. The presence of pain may not always be a reason to rise fear and to avoid activities. Morley et al. (2005) showed that fear is more common when the meaning attached to pain is negative and the individual considers their future self to be conditional on the presence of pain. These patients may have been more stubborn in their maladaptive thoughts and behaviour, or were unable to reflect their cognitions due to underlying psychiatric comorbidities. Therefore, it could be possible that for this heterogenic group EXP is not sufficient and these patients require a more extensive or different treatment approach [62,63].

No moderating factors were revealed, except for gender. Men’s fear levels benefited more from EXP than women’s. Noteworthy, they had higher baseline fear. This is in contrast with the fact that women are more likely to have higher fear levels [64], but it could be ascribed to the small sample size. Patients with higher fear levels were assumed to benefit more from EXP, but no moderator could not demonstrate that. However, it is worth mentioning that all patients already had elevated levels as they were referred to EXP based on the presence of pain-related fear and worries. In addition, no differences between patients with CRPS-I and cLBP were found. This suggests that they did not respond differently to EXP, and conclusions could be applicable for both populations. It has been stated that both populations have similar levels of fear, pain, and disability [12]. Previous research found fast improvement of fear by EXP in patients with cLBP emphasizing insight learning [65], but more gradual progression by trial-and-error learning in patients with CRPS-I [18]. However, our study did not reveal differences in response to EXP based on population.

It is noteworthy that the Body Mass Index of these patients was rather high. Obesity can interact with disability, whether as cause or result [66]. This could have an impact on treatment effects. The fact that exercise programs are able to reduce pain in patients with cLBP and overweight [67] raises the question whether increased activity could explain at least part of the effects. Furthermore, this factor may explain why some patients do not benefit from EXP alone, as obesity requires specific treatment as well [68].

### Strengths and Limitations

One of the strengths of this SCED study is the high number of examined cases. Twenty patients filled out daily diaries with a total of 1136 observations, while previous research established that most SCED studies have an average of three to four cases [69]. Daily diaries were not only interpreted by visual graphs, but also by individual analyses, MCID calculations and multilevel modelling. However, this study also has some limitations. First, the baseline period was mostly short and unstable and not fully randomized (rather the practical context resulted in pseudo-randomization). Second, this study did not include long-term data. Therefore, we cannot evaluate long-term effects. Third, although at least 20 observations per case were collected to prevent biased intervention effects [43], diary completion was rather low. Fourth, interpretation of the MCID should be approached with caution. The cut-off value was determined by three successive measurements, but because of enormous fluctuations later increases are possible. Fifth and last, although the internal validity of SCED studies is strong [43], conclusions should be generalized to the total population with cautiousness. Future research should synthesize information obtained from multiple SCED studies and multiple variables to increase the external validity, especially for identifying treatment moderators.

## 5. Conclusions

The overall findings of this SCED study indicate that EXP reduced pain-related fear as well as pain intensity in patients with cLBP and CRPS-I. However, not all patients responded similarly and different patterns of treatment responses were identified. On an individual level, a reduction in fear was seen in most cases, prior to or in absence of a reduction in pain. For most patients, fear reduced already in the early stage of EXP, and it seemed that fear reductions are necessary to achieve pain relief. The idea that reductions in fear might be necessary to lower pain should encourage clinicians to target fear during rehabilitation. Future research should examine long term effects and should further unravel the benefits of patient clustering for screening and treatment approaches.

## Figures and Tables

**Table 1:** Diary questions.

Topic	Question	Scale
Pain-related fear	“How threatening would it be for you to perform this activity at this moment?” (one question for each of the three tailored activities)	0 = not at all–100 = very
Pain intensity	“How intense is your pain at this moment?” “How intense was your most intense pain during the last 24 h?” “How intense was your least intense pain during the last 24 h?”	0 = not at all–100 = worst imaginable

**Table 2:** Baseline patient characteristics.

Case	Population	Age	Sex (% M)	BMI	Duration Complaints (Total = Median)	Average Pain Score	PDI	PHODA
C01	CRPS LE	43	M	27.47	3–6 m	6.4	34	45.00
C02	CLBP	55	F	26.29	>5 y	5	28	49.43
C03	CLBP	41	F	25.71	2–5 y	6.8	50	61.30
C04	CRPS UE	28	M	24.22	1–2 y	7.6	57	73.00
C05	CLBP	35	M	28.40	2–5 y	3.4	7	18.28
C06	CLBP	36	M	28.70	6–12 m	7	48	43.38
C07	CLBP	28	F	25.00	2–5 y	3.8	41	40.25
C08	CLBP	23	M	22.64	>5 y	7.6	45	71.15
C09	CLBP	37	M	20.28	2–5 y	4	19	28.20
C10	CLBP	53	M	26.85	2–5 y	5.8	39	65.35
C11	CLBP	32	M	29.39	1–2 y	5.6	38	35.38
C12	CLBP	57	M	25.00	2–5 y	4.6	49	71.28
C13	CLBP	52	M	30.76	1–2 y	5.2	35	40.55
C14	CLBP	40	M	29.39	>5 y	3.6	19	54.00
C15	CRPS LE	33	F	27.76	6–12 m	6.2	42	47.95
C16	CLBP	44	M	27.93	>5 y	0	57	70.40
C17	CRPS LE	62	F	31.99	>5 y	6.4	44	83.88
C18	CRPS LE	27	F	24.81	2–5 y	3.6	35	7.28
C19	CRPS UE	34	F	44.63	3–6 m	8.4	49	72.17
C20	CRPS LE	29	F	37.11	1–2 y	8	29	25.25
**Mean CLBP**	13	41	77%	27	2–5 y	5	37	50
**Mean CRPS**	7	37	29%	31	1–2 y	7	41	51
**Overall mean**	20	39	60%	28	2–5 y	5	38	50

CLBP = chronic low back pain; CRPS = complex regional pain syndrome type I; F = female; LE = lower extremities; M = male; PDI = Pain Disability Index (0–70); UE = upper extremities.

**Table 3:** Completeness of daily measurements.

Case	Duration Baseline		Duration EXP		Duration Post-Intervention	
	Duration (Days)	Measurements	Duration (Days (Weeks))	Completion (%)	Duration (Days)	Completion (%)
C01	23	12	94 (14)	100	19	100
C02	9	9	72 (11)	90	12	61
C03	34	9	47 (7)	64	17	56
C04	33	10	66 (10)	41	19	61
C05	7	7	30 (5)	67	0	-
C06	33	10	29 (5)	79	9	89
C07	26	5	59 (9)	66	0	-
C08	35	6	43 (7)	53	0	-
C09	27	11	37 (6)	97	7	89
C10	34	11	66 (10)	55	4	56
C11	47	11	45 (7)	56	0	-
C12	42	11	51 (8)	64	0	-
C13	93	10	57 (9)	88	7	86
C14	31	5	52 (8)	88	0	-
C15	9	9	96 (14)	81	0	-
C16	41	5	57 (9)	81	0	-
C17	52	3	92 (14)	89	12	100
C18	29	9	57 (9)	81	0	-
C19	113	10	52 (8)	51	0	-
C20	65	9	57 (9)	59	0	-
Average	39.15	8.60	57.95 (8.95)	72.50	5.30	77.55

## Data Availability

The datasets generated for this study are available on reasonable request to the responding author.

## Supplementary Materials

The following supporting information can be downloaded at: https://www.mdpi.com/article/10.3390/jcm11051360/s1, Table S1: Multilevel modelling results of the pre-post EXP comparison; Table S2: Individual regression results per week for pain-related fear; Table S3: Individual regression results per week for pain intensity; Table S4A–C: results of non-daily questionnaires; Table S5: Moderating factors in multilevel pre-post model.
