# Supplementary material for: Individual Patterns and Temporal Trajectories of Changes in Fear and Pain during Exposure In Vivo: A Multiple Single-Case Experimental Design in Patients with Chronic Pain
Source: J Clin Med. 2022 Mar 1;11(5):1360. doi: 10.3390/jcm11051360 (PMC8911417; doi:10.3390/jcm11051360)
Supplement: Supplementary file 1 [file jcm-11-01360-s001.zip › jcm-1576761-supplementary.pdf]

Article

Individual Patterns and Temporal Trajectories of Changes in Fear and Pain during Exposure In Vivo: A Multiple Single-Case Experimental Design in Patients with Chronic Pain

Jente Bontinck [1,2], Marlies den Hollander [3,4], Amanda L. Kaas [5], Jeroen R. De Jong [3,6] and Inge Timmers [2,3,*]

Supplementary tables

Table S1: Multilevel modelling results of the pre-post EXP comparison;
Table S2; Individual regression results per week for pain-related fear;
Table S3; Individual regression results per week for pain intensity;
Table S4A-C: results of non-daily questionnaires;
Table S5; Moderating factors in multilevel pre-post model.

Table S1. Multilevel modelling results of the pre-post EXP comparisons.

Fixed effects	Pain-related fear (SD)	Pain intensity (SD)
Intercept (1)	53.17 (6.71)***	42.65 (5.17)***
Time (2)	-0.39 (0.11)**	-0.16 (0.10)
Phase (3)	-29.44 (7.30)***	-9.28 (2.61)**
Time x phase (4)	0.34 (0.33)	0.24 (0.29)

* = $p < 0.05$; ** = $p < 0.01$; *** = $p < 0.001$; 1 = mean baseline level; 2 = mean change in baseline phase; 3 = mean pre-post treatment effect; 4 = mean slope in post-treatment phase; SD = standard deviation.

Table S2. Individual regression results per week for pain-related fear.

Case	Pre / post	Week 1	Week 2	Week 3	Week 4	Week 5	Week 6	Week 7	Week 8	Week 9	Week 10	Week 11	Week 12	Week 13	Week 14	Week 15	Week 16
**C01**	< .001*	0.01*	< .001*	< .001*	< .001*	< .001*	0.015*	0.901	0.005*	< .001*	< .001*	< .001*	0.233	< .001*	< .001*	0.486	0.486
**C02**	< .001*	0.344	< .001*	< .001*	< .001*	< .001*	< .001*	< .001*	< .001*	< .001*	< .001*	0.003*	0.719	0.94	-	-	-
**C03**	0.001*[1]	< .001*	< .001*	< .001*	< .001*	< .001*[1]	< .001*[1]	< .001*[1]	< .001*[1]	0.003*[1]	0.055	0.116	0.868	0.559	0.559	-	-
**C04**	< .001*	0.204	0.078*	< .001*	< .001*	< .001*	< .001*	< .001*	< .001*	0.112	0.112	0.425	0.435	-	-	-	-
**C05**	< .001*	0.596	0.017*	0.703	0.647	-	-	-	-	-	-	-	-	-	-	-	-
**C06**	< .001*	< .001*	< .001*	0.049*	0.938	0.255	0.069	-	-	-	-	-	-	-	-	-	-
**C07**	0.028*	0.018*	0.008*	0.381	0.842	0.415	0.295	0.751	0.757	-	-	-	-	-	-	-	-
**C08**	0.911	0.985	0.001*	0.001*	0.001*	0.141	0.683	-	-	-	-	-	-	-	-	-	-
**C09**	< .001*	< .001*	< .001*	0.587	0.105	0.421	0.942	-	-	-	-	-	-	-	-	-	-
**C10**	< .001*	< .001*	< .001*	< .001*	< .001*	< .001*	< .001*	< .001*	0.03*	0.819	0.819	-	-	-	-	-	-
**C11**	0.005*	0.403	0.221	0.304	0.62	0.041*	0.176	0.589	-	-	-	-	-	-	-	-	-
**C12**	0.383	0.346	0.107	0.097	0.027*	0.009*	0.024*	0.013*	-	-	-	-	-	-	-	-	-
**C13**	< .001*	0.672	< .001*	< .001*	< .001*	0.004*	0.025*	0.098	0.364	0.946	-	-	-	-	-	-	-
**C14**	0.001*	0.991	0.037*	0.073	0.556	0.151	0.294	0.933	-	-	-	-	-	-	-	-	-
**C15**	0.182	0.001*	0.006*	< .001*	< .001*	0.012*	0.001*	< .001*	< .001*	< .001*	0.002*	0.024*	0.292	0.614	-	-	-
**C16**	< .001*	0.979	0.149	0.55	0.644	0.259	0.263	0.561	0.822	-	-	-	-	-	-	-	-
**C17**	< .001*	0.812	0.963	0.55	0.014*	< .001*	< .001*	< .001*	< .001*	0.001*	0.067	0.073	0.486	0.645	-	-	-
**C18**	0.21	< .001*	< .001*	< .001*	< .001*	< .001*	0.034*	0.17	0.608	-	-	-	-	-	-	-	-
**C19**	0.518	0.28	0.014*	0.043*	0.007*	0.448	0.858	0.107	-	-	-	-	-	-	-	-	-
**C20**	0.653	0.554	0.51	0.71	0.052	0.992	0.278	0.841	0.536	-	-	-	-	-	-	-	-

* = p-value < 0.05; [1] = increase instead of reduction.

Table S3. Individual regression results per week for pain intensity.

Case	Pre / post	Week 1	Week 2	Week 3	Week 4	Week 5	Week 6	Week 7	Week 8	Week 9	Week 10	Week 11	Week 12	Week 13	Week 14	Week 15	Week 16
**C01**	< .001*	0.026*	< .001*	< .001*	< .001*	< .001*	< .001*	< .001*	< .001*	< .001*	< .001*	0.002*	0.233	0.524	0.818	0.913	0.913
**C02**	0.005*	0.003*	< .001*	< .001*	< .001*	0.005*	0.016*	0.099	0.777	0.134	0.115	0.082	0.491	0.592	-	-	-
**C03**	0.503	0.031*	0.067	0.716	0.6	0.451	0.319	0.513	0.275	0.38	0.619	0.722	0.868	0.952	0.952	-	-
**C04**	< .001*	0.072	0001*	0.004*	0.003*	0.034	0.794	0.03*	0.03*	0.15	0.15	0.366	0.435	-	-	-	-
**C05**	0.43	0.56	0.798	0.609	0.963	-	-	-	-	-	-	-	-	-	-	-	-
**C06**	0.447	0.831	0.851	0.618	0.015*	0.786	0.393	-	-	-	-	-	-	-	-	-	-
**C07**	0.076*	0.665	0.899	0.714	0.638	0.878	0.597	0.382	0.052	-	-	-	-	-	-	-	-
**C08**	0.125	0.104	0.811	0.998	0.779	0.68	0.114	-	-	-	-	-	-	-	-	-	-
**C09**	0.528	0.02*	0.099	0.68	0.614	0.428	0.84	-	-	-	-	-	-	-	-	-	-
**C10**	0.961	< .001*	< .001*	< .001*	< .001*	< .001*	0.005*	0.274	0.214	0.986	0.986	-	-	-	-	-	-
**C11**	0.014*	0.463	0.054	0.26	0.989	0.052	0.458	0.455	-	-	-	-	-	-	-	-	-
**C12**	0.756	0.528	0.202	0.492	0.763	< .001*	0.001*	0.013*	-	-	-	-	-	-	-	-	-
**C13**	0.059	0.027*	0.004*	< .001*	< .001*	0.002*	0.081	0.36	0.706	0.364	-	-	-	-	-	-	-
**C14**	0.083	0.697	0.388	0.299	0.406	0.439	0.094	0.681	-	-	-	-	-	-	-	-	-
**C15**	0.071	0.003*	0.165	0.884	0.754	0.067	0.009*	0.001*	0.072	0.709	0.146	0.038*	0.292	0.068	-	-	-
**C16**	0.372	0.022*	< .001*	< .001*	< .001*	< .001*	0.003*	0.149	0.718	-	-	-	-	-	-	-	-
**C17**	0.512	< .001*	0.839	0.696	0.631	0.659	0.891	0.779	0.745	0.699	0.043	0.352	0.486	0.529	-	-	-
**C18**	0.961	0.114	0.21	0.583	0.516	0.001*	0.018*	0.061	0.707	-	-	-	-	-	-	-	-
**C19**	0.92	0.241	0.075	0.008	0.002*	0.124	0.109	0.344	-	-	-	-	-	-	-	-	-
**C20**	0.006*[1]	0.051*[1]	0.012*[1]	0.025*[1]	0.016*[1]	0.425	0.578	0.812	0.61	-	-	-	-	-	-	-	-

* = p-value < 0.05; [1] = increase instead of reduction.

Table S4A. Results of non-daily questionnaires.

Pre-EXP vs post-EXP	Mean (SD)	SE	95% CI	t-value	p-value
Average pain	-1.64 (2.16)	.48	.63 – 2.64	3.40	.003*
PDI	-27.40 (15.36)	3.43	20.21 – 34.59	7.98	< .001*
PHODA	-33.32 (22.69)	5.07	22.71-43.95	6.57	< .001*
TSK	-10.65 (7.39)	1.65	7.19 – 14.11	6.45	< .001*
HADS	-2.35 (7.17)	1.60	-1.01 – 5.71	1.47	.159
PCS	-12.40 (13.08)	2.92	6.28 - 18.52	4.24	< .001*
PVAQ	-13.75 (16.57)	3.70	5.99 – 21.51	3.71	.001*
SFMPQ	-5.40 (6.48)	1.45	2.37 – 8.43	3.73	.001*
RS	-7.10 (7.45)	1.67	3.61 – 10.59	4.26	< .001*

* = p-value < 0.05.

Table S4B. Results of non-daily questionnaires.

Case	Average pain score				PDI				PHODA				TSK				HADS angst + depressie			
	PRE	POST	DIFF	%	PRE	POST	DIFF	%	PRE	POST	DIFF	%	PRE	POST	DIFF	%	PRE	POST	DIFF	%
**CLUSTER 1**	**4.5**	**2.1**	**2.0**	**48%**	**37.1**	**4.3**	**32.9**	**85%**	**53.6**	**6.2**	**47.4**	**88%**	**35.1**	**24.9**	**10.3**	**27%**	**6.3**	**4.9**	**1.4**	**-**
C01	3.4	0	3.4	100%	34	0	34	100%	45	0.1	45.0	100%	27	25	2	7%	0	1	-1	-
C02	6.2	5	1.2	19%	28	10	18	64%	49.43	18.9	30.6	62%	26	23	3	12%	0	13	-13	-
C04	7	3.8	3.2	46%	57	0	57	100%	73	0.4	72.6	99%	39	29	10	26%	0	3	-3	-
C07	3.4	2	1.4	41%	41	6	35	85%	40.3	0.0	40.3	100%	26	21	5	19%	18	2	16	89%
C10	3.2	3.2	0	0%	39	7	32	82%	65.4	9.5	55.9	85%	39	18	21	54%	5	6	-1	-20%
C14	3	0.4	2.6	87%	19	7	12	63%	54	7.4	46.6	86%	51	37	14	27%	11	6	5	45%
C15	5.6	3.2	2.4	43%	42	0	42	100%	48	6.9	41.1	86%	38	21	17	45%	10	3	7	70%
**CLUSTER 2**	**4.8**	**3.2**	**1.6**	**26%**	**30.6**	**11.4**	**19.2**	**63%**	**42.9**	**12.4**	**30.5**	**82%**	**38.8**	**27.2**	**11.6**	**29%**	**10.4**	**9.2**	**1.2**	**5%**
C05	4	2.2	1.8	45%	7	4	3	43%	18.3	0.9	17.4	95%	36	32	4	11%	7	10	-3	-43%
C06	4	6.6	-2.6	-65%	48	9	39	81%	43.4	0.0	43.4	100%	46	21	25	54%	4	4	0	0%
C09	5	0	5	100%	19	0	19	100%	28.2	0.0	28.2	100%	42	29	13	31%	10	6	4	40%
C13	3.8	3	0.8	21%	35	15	20	57%	40.6	9.0	31.6	78%	37	30	7	19%	21	17	4	19%
C17	7	4	3	43%	44	29	15	34%	83.9	52.1	31.8	38%	33	24	9	27%	10	9	1	10%
**CLUSTER 3**	**4.9**	**2.5**	**2.4**	**26%**	**46.7**	**9.3**	**37.3**	**80%**	**59**	**9.78**	**49.20**	**84%**	**46**	**34.3**	**11.7**	**23%**	**14.3**	**4.7**	**9.7**	**59%**
C08	7.6	6.8	0.8	11%	45	7	38	84%	71.2	20.0	51.2	72%	32	29	3	9%	8	5	3	38%
C11	7.2	0.8	6.4	89%	38	9	29	76%	35.4	3.8	31.6	89%	44	30	14	32%	23	4	19	83%
C16	0	0	0	0%	57	12	45	79%	70.4	5.5	64.9	92%	62	44	18	29%	12	5	7	58%
**CLUSTER 4**	**5.8**	**5.1**	**0.7**	**21%**	**42.4**	**20.4**	**22**	**51%**	**47.5**	**40.5**	**6.9**	**33%**	**36.8**	**27.2**	**9.6**	**24%**	**9.8**	**9.4**	**0.4**	**-23%**
C03	2.8	0	2.8	100%	50	0	50	100%	61.3	93.6	-32.3	-53%	50	25	25	50%	14	7	7	50%
C12	5.2	7.8	-2.6	-50%	49	25	24	49%	71.2	52.7	18.6	26%	40	35	5	13%	11	15	-4	-36%
C18	6.2	5.4	0.8	13%	35	10	25	71%	7.2	0.4	6.9	95%	23	18	5	22%	1	2	-1	-100%
C19	7.8	6.4	1.4	18%	49	47	2	4%	72.2	46.4	25.8	36%	36	27	9	25%	15	10	5	33%
C20	7	6	1	14%	29	20	9	31%	25.3	9.65	15.6	62%	35	31	4	11%	8	13	-5	-63%
**TOTAAL**	**5**	**3.3**	**1.6**	**34%**	**38.3**	**10.9**	**27.4**	**70%**	**50.2**	**16.9**	**33.3**	**72%**	**38.1**	**27.5**	**10.7**	**26%**	**9.4**	**7.1**	**2.3**	**-**

Pre = baseline results; Post = post-treatment results; Diff = improvement by treatment; % = percentage improvement by treatment.

Table S4C. Results of non-daily questionnaires.

Case	PCS				PVAQ				SFMPQ				RS			
	PRE	POST	DIFF	%	PRE	POST	DIFF	%	PRE	POST	DIFF	%	PRE	POST	DIFF	%
**CLUSTER 1**	**15**	**4.9**	**10.1**	**48%**	**43**	**23**	**20**	**43%**	**15.1**	**9.7**	**5.4**	**28%**	**47.9**	**36**	**11.9**	**24%**
C01	12	0	12	100%	37	34	3	8%	12	1	11	92%	49	35	14	29%
C02	12	4	8	67%	32	19	13	41%	14	14	0	0%	43	30	13	30%
C04	30	0	30	100%	62	3	59	95%	19	3	16	84%	51	28	23	45%
C07	26	3	23	88%	43	9	34	79%	19	20	-1	-5%	51	36	15	29%
C10	3	5	-2	-67%	31	17	14	45%	7	12	-5	-71%	44	42	2	5%
C14	15	21	-6	-40%	39	33	6	15%	13	6	7	54%	57	46	11	19%
C15	7	1	6	86%	57	46	11	19%	22	12	10	45%	40	35	5	13%
**CLUSTER 2**	**23.8**	**6.8**	**17**	**55%**	**41.4**	**28.8**	**12.6**	**25%**	**16.8**	**13.4**	**3.4**	**18%**	**46**	**40.6**	**5.4**	**10%**
C05	12	16	-4	-33%	33	37	-4	-12%	12	10	2	17%	43	38	5	12%
C06	32	2	30	94%	39	18	21	54%	18	14	4	22%	41	33	8	20%
C09	39	0	39	100%	55	10	45	82%	13	13	0	0%	39	28	11	28%
C13	21	10	11	52%	38	42	-4	-11%	22	15	7	32%	56	53	3	5%
C17	15	6	9	60%	42	37	5	12%	19	15	4	21%	51	51	0	0%
**CLUSTER 3**	**23**	**12**	**11**	**61%**	**25**	**21**	**4**	**19%**	**18.33**	**8.7**	**9.7**	**58%**	**51**	**41.7**	**9.3**	**5%**
C08	4	0	4	100%	25	16	9	36%	15	4	11	73%	33	33	0	0%
C11	35	6	29	83%	21	13	8	38%	16	3	13	81%	66	50	16	24%
C16	30	30	0	0%	29	34	-5	-17%	24	19	5	21%	54	42	12	22%
**CLUSTER 4**	**22**	**10.2**	**11.8**	**54%**	**39.4**	**27.4**	**12**	**31%**	**19**	**14.2**	**4.8**	**22%**	**45.8**	**45**	**0.8**	**3%**
C03	32	18	14	44%	37	15	22	59%	17	0	17	100%	35	38	-3	-9%
C12	16	18	-2	-13%	60	55	5	8%	14	21	-7	-50%	48	54	-6	-13%
C18	7	1	6	86%	29	19	10	34%	16	15	1	6%	51	46	5	10%
C19	35	10	25	71%	42	19	23	55%	26	18	8	31%	52	42	10	19%
C20	20	4	16	80%	29	29	0	0%	22	17	5	23%	43	45	-2	-5%
**TOTAAL**	**20.2**	**7.8**	**12.4**	**53%**	**39**	**25.3**	**13.8**	**32%**	**17**	**11.6**	**5.4**	**29%**	**47.4**	**40.3**	**7.1**	**14%**

Pre = baseline results; Post = post-treatment results; Diff = improvement by treatment; % = percentage improvement by treatment.

Table S5. Moderating factors in multilevel pre-post model.

	Fixed effects	Pain-related fear (SD)	Pain intensity (SD)		Fixed effects	Pain-related fear (SD)	Pain intensity (SD)		Fixed effects	Pain-related fear (SD)	Pain intensity (SD)
**Gender**	Factor (1)	-23.29 (10.01)*	7.36 (8.77)	**PDI**	Factor (1)	0.95 (0.38)*	0.34 (0.33)	**PVAQ**	Factor (1)	0.39 (0.45)	0.10 (0.36)
	Phase x factor (2)	37.17 (10.26)**	2.53 (5.62)		Phase x factor (2)	0.17 (0.52)	-0.11 (0.21)		Phase x factor (2)	-0.24 (0.54)	-0.27 (0.22)
**Age**	Factor (1)	0.74 (0.44)	-0.20 (0.37)	**TSK**	Factor (1)	0.77 (0.57)	-0.52 (0.45)	**SFMPQ**	Factor (1)	0.62 (1.20)	0.30 (0.94)
	Phase x factor (2)	-0.14 (0.56)	-0.17 (0.23)			-0.21 (0.70)	-0.51 (0.27)		Phase x factor (2)	0.73 (1.41)	0.22 (0.60)
**Group**	Factor (1)	4.44 (11.70)	-13.47 (8.62)	**PCS**	Factor (1)	0.58 (0.46)	-0.01 (0.38)	**RS**	Factor (1)	-0.23 (0.56)	-0.23 (0.56)
	Phase x factor (2)	-5.26 (13.78)	-5.33 (5.66)		Phase x factor (2)	-0.07 (0.57)	0.09 (0.24)		Phase x factor (2)	0.13 (0.35)	0.13 (0.35)
**PHODA**	Factor (1)	0.78 (0.20)*	0.21 (0.21)	**HADS**	Factor (1)	0.32 (0.85)	-0.24 (0.66)				
	Phase x factor (2)	0.09 (0.32)	-0.17 (0.13)		Phase x factor (2)	0.87 (0.98)	-0.41 (0.40)				

* = $p < 0.05$; ** = $p < 0.01$; *** = $p < 0.001$; 1 = influence of factor on baseline scores; 2 = influence of factor on treatment effect; HADS = Hospital Anxiety and Depression Scale; PCS = Pain Catastrophizing Scale; PDI = Pain Disability Index; PHODA = Photographs series of Daily Activities; PVAQ = Pain Vigilance and Awareness Questionnaire; RS = Resilience Scale; SD = standard deviation; SFMPQ = Short-Form McGill Pain Questionnaire; TSK = Tampa Scale for Kinesiophobia.
